# A genome-wide association analysis for porcine serum lipid traits reveals the existence of age-specific genetic determinants

**DOI:** 10.1186/1471-2164-15-758

**Published:** 2014-09-04

**Authors:** Arianna Manunza, Joaquim Casellas, Raquel Quintanilla, Rayner González-Prendes, Ramona N Pena, Joan Tibau, Anna Mercadé, Anna Castelló, Nitdia Aznárez, Jules Hernández-Sánchez, Marcel Amills

**Affiliations:** Department of Animal Genetics, Center for Research in Agricultural Genomics (CSIC-IRTA-UAB-UB), Universitat Autònoma de Barcelona, Bellaterra, 08193 Spain; Departament de Ciència Animal i dels Aliments, Universitat Autònoma de Barcelona, Bellaterra, 08193 Spain; IRTA, Genètica i Millora Animal, Lleida, 25198 Spain; Departament de Producció Animal, Agrotecnio Center, Universitat de Lleida, Lleida, 25198 Spain; IRTA, Finca Camps i Armet, 17121 Monells, Spain

**Keywords:** Animal model, Lipids and lipoproteins, Genome-wide analysis, Gene expression

## Abstract

**Background:**

The genetic determinism of blood lipid concentrations, the main risk factor for atherosclerosis, is practically unknown in species other than human and mouse. Even in model organisms, little is known about how the genetic determinants of lipid traits are modulated by age-specific factors. To gain new insights into this issue, we have carried out a genome-wide association study (GWAS) for cholesterol (CHOL), triglyceride (TRIG) and low (LDL) and high (HDL) density lipoprotein concentrations measured in Duroc pigs at two time points (45 and 190 days).

**Results:**

Analysis of data with mixed-model methods (EMMAX, GEMMA, GenABEL) and PLINK showed a low positional concordance between trait-associated regions (TARs) for serum lipids at 45 and 190 days. Besides, the proportion of phenotypic variance explained by SNPs at these two time points was also substantially different. The four analyses consistently detected two regions on SSC3 (124 Mb, CHOL and LDL at 190 days) and SSC6 (135 Mb, CHOL and TRIG at 190 days) with highly significant effects on the porcine blood lipid profile. Moreover, we have found that SNP variation within SSC3, SSC6, SSC10, SSC13 and SSC16 TARs is associated with the expression of several genes mapping to other chromosomes and related to lipid metabolism.

**Conclusions:**

Our data demonstrate that the effects of genomic determinants influencing lipid concentrations in pigs, as well as the amount of phenotypic variance they explain, are influenced by age-related factors.

**Electronic supplementary material:**

The online version of this article (doi:10.1186/1471-2164-15-758) contains supplementary material, which is available to authorized users.

## Background

Understanding the genetic architecture of blood lipids from an evolutionary perspective will be only feasible through the genetic analysis of multiple species. Comparative studies performed in mouse for HDL concentrations have highlighted that there are remarkable concordances between human and murine QTL maps [1] as well as between QTL (mouse) and GWAS (human) trait-associated regions (TARs) [2]. These results suggest that lipid homeostasis is regulated by a common set of genes in both species.

Pigs are particularly relevant from a clinical point of view because they develop atherosclerotic vascular lesions that are similar to those observed in humans [3]. There is substantial evidence that additive variance for serum lipid levels exists in swine populations, with heritability estimates that range between 0.2-0.4 [4]. Besides, several porcine serum lipid QTL have been detected with the aid of microsatellite markers [5–8] and, more recently, it was reported the first GWAS for porcine blood lipid traits in Duroc x Erhualian F_2_ and Sutai pigs [9]. As in humans, GWAS approaches should be extended to a large number of pig populations in order to achieve a comprehensive and reliable picture of genomic variation affecting serum lipid concentrations.

In the current work, we aimed to investigate the genetic architecture of total cholesterol (CHOL), low (LDL) and high (HDL) density lipoprotein and triglyceride (TRIG) concentrations in serum samples obtained from Duroc pigs at two different ages (45 and 190 days). Previous heritability data obtained in humans [10] and pigs [11] suggested that the genetic determinism of lipid concentrations varies with age, so our main goal was to evaluate the positional concordance of TARs determining porcine serum lipid concentrations at two different timepoints. We have used four statistical packages to carry out GWAS analyses in order to identify those TARs that are consistently detected by all programs.

## Methods

### Phenotyping of a Duroc commercial population

The Duroc resource population employed in the current experiment and phenotyping methods have been reported by Gallardo et al. [5]. Briefly, a commercial Duroc line (Lipgen population) consisting of 350 barrows distributed in five half-sib families was generated by crossing 5 boars with ~400 sows. After weaning, this pig population was transferred to the experimental test station at the *Centre de Control Porcí* (CCP) of the *Institut de Recerca i Tecnologia Agroalimentàries* (IRTA). Serum lipids were measured in 45 and 190 days-old pigs by following the protocols reported by Gallardo et al. [5]. Data (CHOL45, CHOL190, LDL45, LDL190, HDL45, HDL190, TRIG45 and TRIG190) were log-transformed prior to GWAS analyses in order to ensure normality [5]. The experimental procedures, traits recording and blood sampling were approved by the IRTA Ethical Committee.

### Measurement of hepatic gene expression phenotypes

Global mRNA expression datasets were generated for liver samples obtained from 97 Duroc pigs from the Lipgen population (our unpublished data). Total RNA was isolated and hybridised to GeneChip Porcine arrays (Affymetrix Inc., Santa Clara, CA) as previously reported [12]. Microarray data were normalised with the gcRMA algorithm, using the BRB-ArrayTools software version 3.7.1 [13] and deposited in the Gene Expression Omnibus (GEO) public repository (GSE19275, GSE26091 and GSE48992 accession numbers). Probes corresponding to 16,949 genes were obtained with the most updated annotation file [14].

### High throughput genotyping and data filtering

DNA samples were genotyped using the Illumina Infinium HD Porcine SNP60 Beadchip (Illumina, San Diego, CA) according to the manufacturer instructions (http://www.illumina.com). Quality genotyping analyses were performed with the GenomeStudio software (Illumina). The GenCall score cut-off and the average call rate were 0.15 and 97%, respectively. Single nucleotide polymorphisms with a rate of missing genotypes > 5%, that did not conform Hardy-Weinberg expectations (threshold set at a *P*-value = 0.001) or that had a minor allele frequency below 0.05 were eliminated from the dataset. SNPs mapping to the X chromosome were also excluded from the analyses. After frequency and genotype pruning, the final dataset included a total of 37,960 SNPs and 320 individuals.

### Genome-wide association analyses

The statistical models employed in the analysis of the serum lipid data were as follows:


where y_*i*_ is the phenotypic record (CHOL, LDL, HDL and TRIG at 45 or 190 days of age) collected from the *i*th individual; μ is the mean of the serum lipid trait in the population; *batch* and *farm* are the systematic effects *i.e.* batch of fattening (with 4 categories) and farm of origin (with 3 categories); *cov*_*i*_ is a covariate that depends on the trait (live weight at slaughter for CHOL, HDL and LDL; age at slaughter for TRIG); β_*cov*_ and β_g_ are the partial regression coefficients of *y*_*i*_ on *cov*_*i*_ and of *y*_*i*_ on *g*_*i*_, respectively; *g*_*i*_ represents the SNP genotype and *e*_*i*_ is the error associated with the model. The statistical relevance of the systematic environmental sources of variation and the covariates were previously corroborated by Gallardo et al. [5] and Casellas et al. [11] on the same dataset.

Gene expression data were exclusively analysed with GEMMA. The statistical model took into consideration the fixed effects “batch of fattening” (with four categories) and “laboratory” (with 2 levels, since microarray data were generated in two different laboratories). Since this type of study involves the performance of 8.59 million tests (507 SNPs × 16,949 probes) we used the Bonferroni correction to take into account multiple testing (threshold of significance: 1.16 × 10e-7).

#### PLINK analysis of the data

We made a first analysis of the data with the PLINK toolset [15]. This package has been mostly used in the framework of case/control studies, but it also allows the analysis of quantitative traits through standard linear regression. PLINK also offers a variety of methods to take into account population stratification, but other sources of sample structure, such as hidden relatedness, are more difficult to correct with these tools [15, 16]. Although PLINK supports family-based association analysis based on transmission disequilibrium testing (QFAM), which is particularly robust to the effects of sample structure, we decided not to use this approach because only sire and offspring (1 per sow) genotypes, but not the mother’s ones, were available in the Lipgen population. Thus, we made a first analysis without taking into account sample structure. We retrieved all the significant SNPs (*P*-value < 0.05 after correction for multiple testing with a false discovery rate approach) and they were used in a second round of analysis where sires were considered as a fixed effect (with 5 levels). In principle, this approach considers family-specific genetic structure by taking into account sire-related genetic effects. However, we did not expect any bias produced by sows because the ~ 400 females belonging to the Lipgen population were randomly mated with the 5 parental boars.

#### Analysis of the data with mixed-model statistical packages

Three mixed-effects models were used in addition to PLINK to estimate the robustness of the associations found. A brief description of these programs follows. The Efficient Mixed-Model Association eXpedited (EMMAX) package [16] builds a pairwise relatedness matrix on the basis of SNP genotypes and, subsequently, a variance component model is used to infer the contribution of sample structure to phenotypes. This is achieved by constructing a covariance matrix of phenotypes that represents the effects of genetic relatedness on phenotypes [16]. Associations between SNPs and traits are tested applying a correction for sample structure (population stratification and hidden relatedness) through the covariance matrix.

The Genome-wide Rapid Association using Mixed Model and Regression (GRAMMAR) approach implemented in GenABEL was also used to carry out GWAS [17]. GRAMMAR infers pairwise kinship coefficients amongst sampled individuals on the basis of genomic marker data. Then, additive polygenic effects are estimated, adjusting for fixed (nuisance) effects, and the residuals are used in a second step as phenotypes in GWAS [17]. Finally, we also used the Genome-wide Efficient Mixed-Model Association (GEMMA) approach developed by Zhou and Stephens [18], that also uses a standard linear mixed model to account for sample structure but, in contrast with the two preceding approximate methods, provides an exact test for significance. GEMMA was also employed to estimate the proportion of phenotypic variance explained by SNP genotypes (*i.e.* “chip heritability”), which can be summarized as follows:


where , is the variance due to markers and  is the residual variance.

Correction for multiple testing was implemented with the Bonferroni method as well as with the false discovery rate approach [19].

#### Analysing the gene content of trait-associated regions

Genes mapping to TARs were retrieved from the Ensembl database with the Biomart data mining tool [20] and mapped (PLINK analysis) to the Reactome database [21]. Orthologous relationships between pig and human TARs were inferred with the aid of the National Human Genome Research Institute (NHGRI) GWAS Catalog [22] database (http://www.genome.gov/gwastudies).

## Results

### Phenotypic variance of porcine serum lipid traits explained by the genotyped SNPs

We have estimated the proportion of phenotypic variance (V_P_) explained by all the SNPs (h^2^_SNP_) at the whole-genome level with GEMMA (Table 1). With regard to serum lipids at 45 days, as much as 43% of TRIG45 V_P_ could be attributed to the SNPs genotyped in the Lipgen population, while for CHOL45 and LDL45 this percentage was lower (~13-14%). The proportion of V_P_ corresponding to serum lipids at 190 days explained by the SNPs reached values of 27%, 33% and 19% for CHOL190, LDL190 and TRIG190, respectively. The two phenotypes with the smaller amount of h^2^_SNP_ were HDL45 (0%) and HDL190 (2%). We corroborated these estimates by using the EMMAX and GenABEL softwares (Additional file 1: Figure S1), which yielded similar results *i.e.* very low h^2^_SNP_ for HDL traits and moderate values for the remaining phenotypes.

**Table 1:** **Fraction of the phenotypic variance explained by the markers contained in the Porcine 60 K BeadChip (column 2) compared with Bayesian estimates of heritabilities (columns 3-4) obtained by Casellas et al.** [11]

Trait ^a^	h ^2^_SNP_(GEMMA)	Bayesian h ^2^estimate	BF ^b^
CHOL45	0.14 ± 0.10	0.38 (0.06-0.68)	3.1
LDL45	0.13 ± 0.07	0.27 (0.00-0.62)	1.3
HDL45	0.00 ± 0.08	0.47 (0.01-0.81)	2.2
TRIG45	0.43 ± 0.13	0.42 (0.07-0.70)	8.7
CHOL190	0.27 ± 0.10	0.37 (0.08-0.62)	47.9
LDL190	0.33 ± 0.11	0.36 (0.07-0.60)	16.3
HDL190	0.02 ± 0.04	0.45 (0.02-0.80)	2.1
TRIG190	0.19 ± 0.10	0.34 (0.05-0.58)	16.1

^a^Traits were measured at 45 and 190 days: cholesterol (CHOL), low (LDL) and high (HDL) density lipoproteins and triglycerides (TRIG).

^b^Bayes factor.

### Genomic regions significantly associated with serum lipid concentrations in pigs

We have used GEMMA, EMMAX, GenABEL and PLINK to identify genomic regions displaying significant associations with serum lipids. In general, EMMAX, GEMMA and GenABEL results were very consistent (Table 2), while PLINK identified most of the regions found with the three mixed-effects models plus many others that, with these programs, appeared as non-significant (Table 3 and Additional file 2: Table S1). With GenABEL and GEMMA, we were able to detect two genome-wide significant associations for CHOL190 at SSC3 (124 Mb) and SSC6 (135 Mb). Besides, several chromosome-wide significant associations emerged consistently in the three mixed-model analyses (i) SSC3 (124 Mb) with CHOL190 (it attained genome-wide significance with GenABEL and GEMMA), LDL45 and LDL190; (ii) SSC6 (135 Mb) with CHOL190 (it attained genome-wide significance with GenABEL and GEMMA) and TRIG190; and (iii) SSC16 (17 Mb) with CHOL45 (Table 2). The Manhattan plots of the TARs identified with GEMMA are shown at Additional file 1: Figure S2. Importantly, there was not any positional concordance between regions determining serum lipid concentrations at 45 and 190 days.

**Table 2:** Significant genome-wide (*) and chromosome-wide associations for serum lipid traits detected with three different mixed-model approaches ^**1**^

EMMAX										
Trait	CHR	N	SNP	Reg (Mb)	***P***	q	Bonf.	E	A1	MAF
LDL45	SSC3	3	ASGA0016334	124.6-124.8	2.82x10e-05	0.02	0.05	0.04	A	0.32
LDL190	SSC3	2	ASGA0016334	124.8	6.42x10e-06	0.006	0.02	0.05	A	0.32
CHOL45	SSC16	2	DRGA0015896	17.7	2.64x10e-05	0.01	0.03	-0.05	T	0.12
CHOL190	SSC3	3	ASGA0016334	124.6-124.8	6.82x10e-06*	0.01	0.006	0.03	A	0.32
	SSC6	5	ASGA0029689	135.0-135.4	1.32x10e-05*	0.01	0.03	-0.03	G	0.36
TRIG190	SSC6	4	ASGA0101719	10.2-10.7	6.9x10e-05	0.03	0.1	0.06	G	0.36
		6	ASGA0089937	135.0-135.4	4.48x10e-06	0.006	0.01	0.06	A	0.34
**GEMMA**										
LDL45	SSC3	3	ALGA0021216	124.6-124.8	1.51x10e-05	0.01	0.03	-0.05	A	0.33
	SSC10	5	ASGA0097841	2.5-2.9	1.90x10e-04	0.03	0.25	0.04	G	0.47
		5	MARC0064247	18.2-19.3	1.77x10e-05	0.01	0.02	0.06	A	0.34
	SSC13	1	ALGA0074022	215.0	1.68x10e-05	0.05	0.05	0.09	G	0.07
LDL190	SSC3	2	ALGA0021216	124.8	4.03x10e-06	0.007	0.01	-0.05	A	0.33
CHOL45	SSC3	3	ALGA0021216	124.8-133.5	4.40x10e-05	0.03	0.08	-0.04	A	0.33
	SSC10	2	MARC0064247	19.2	5.73x10e-05	0.04	0.07	0.04	A	0.34
	SSC16	2	ASG A0072378	17.7	1.50x10e-05	0.01	0.02	0.05	G	0.12
CHOL190	SSC3	2	ALGA0021216	124.8	4.53x10e-06*	0.004	0.01	-0.04	A	0.33
	SSC6	5	INRA0022506	135.0-135.4	6.82x10e-06*	0.01	0.02	0.03	A	0.36
TRIG190	SSC6	4	MARC0042729	10.2-10.7	8.68x10e-05	0.03	0.21	-0.07	A	0.36
		1	ASGA0106002	68.1	1.79x10e-04	0.04	0.44	-0.07	A	0.43
		6	ASGA0089937	135.0-135.4	3.95x10e-06	0.01	0.01	-0.07	A	0.35
**GenABEL** ^**2**^										
LDL45	SSC3	2	ASGA0016334	124.8	1.76x10e-05	0.01	0.03	-0.51	A	0.32
	SSC13	1	ALGA0074022	215.0	1.28x10e-05	0.03	0.03	0.94	G	0.06
LDL190	SSC3	2	ASGA0016334	124.8	6.97x10e-06	0.006	0.01	-0.85	A	0.32
CHOL45	SSC16	2	DRGA0015896	17.7	4.06x10e-05	0.02	0.05	0.43	T	0.12
CHOL190	SSC3	2	ASGA0016334	124.8	7.39x10e-06*	0.007	0.01	-0.54	A	0.32
	SSC6	5	ASGA0029689	135.0-135.4	1.26x10e-06*	0.001	0.003	0.58	G	0.36
TRIG45	SSC10	1	MARC0003307	38.0	3.12x10e-05	0.04	0.04	-0.95	A	0.32
TRIG190	SSC6	7	ASGA0089937	135.0-136.1	2.30x10e-06	0.003	0.005	-0.88	A	0.34
HLD45	SSC18	5	ALGA0096968	9.8-12.2	6.68x10e-05	0.03	0.06	-1.74	C	0.21
		2	MARC0006153	20.8-22.6	6.15x10e-05	0.03	0.05	-1.32	A	0.46

^1^ **N:** Number of significant SNPs, CHR: chromosome, SNP: most significant SNP, Reg (Mb): region containing significant SNPs according to Ensembl (*S.scrofa* 10.2), P: nominal *P*-value, q: q-value with FDR ≤ 0.05, Bonf: Bonferroni-corrected P-value, E: allelic effect, A1: minority allele, MAF: frequency of the minority allele.

^2^In GenABEL, allele effects are corrected dividing by the GRAMMAR-gamma factor, thus, their magnitudes are greater than the effects estimated with GEMMA and EMMAX [23].

**Table 3:** **List of the most significant associations detected with PLINK for serum lipid traits** ^**1**^

Trait	CHR	N	SNP	Reg(Mb)	***P***	q	Bonf	E	A1	MAF
**LDL190**	SSC3	13	ALGA0021216	124.8-138.9	1.46x10e-04	0.01	0.03	-0.04	A	0.33
	SSC6	7	ALGA0036903	126.6-135.1	4.46x10e-04	0.01	0.10	0.04	A	0.35
	SSC10	4	ALGA0103072	69.8-76.9	3.62x10e-04	0.01	0.08	0.04	G	0.31
**CHOL190**	SSC1	6	M1GA0001375	264.6-271.9	1.49x10e-04	0.0037	0.03	0.03	A	0.39
	SSC3	5	ALGA0021216	124.8-138.6	2.84x10e-04	0.01	0.06	-0.03	A	0.33
	SSC6	1	ALGA0110498	93.7	1.42x10e-04	0.0037	0.03	-0.04	A	0.12
		7	ALGA0037119	135.0-136.2	4.86x10e-05	0.0035	0.01	0.03	A	0.36
		14	ASGA0030240	145.9-153.4	9.02x10e-05	0.0034	0.02	0.03	A	0.32
	SSC10	2	ALGA0103072	76.8-76.9	3.74x10e-04	0.01	0.09	0.03	G	0.31
**TRIG45**	SSC2	1	MARC0001645	87.1	5.97x10e-04	0.01	0.08	0.04	A	0.33
	SSC7	1	MARC0058691	10.7	1.73x10e-04	0.01	0.02	0.06	G	0.20
		9	H3GA0022580	95.6-99.9	5.61x10e-04	0.01	0.07	0.05	G	0.38
		2	ALGA0043835	100.0-100.1	4.09x10e-04	0.01	0.05	0.05	A	0.38
	SSC10	7	MARC0042485	0.9-16.1	7.04x10e-06	0.00092	0.00092	0.06	A	0.29
		7	MARC0003307	38.0-39.0	1.11x10e-04	0.01	0.01	-0.05	A	0.33
	SSC11	1	H3GA0031439	16.2	4.82x10e-04	0.01	0.06	-0.05	A	0.30
	SSC14	33	ALGA0080030	101.1-114.0	2.05x10e-04	0.01	0.03	-0.07	A	0.14
	SSC16	4	ASGA0072378	4.2-17.7	5.29x10e-04	0.01	0.07	0.06	G	0.12
**TRIG190**	SSC1	4	ALGA0108388	28.6-30.2	3.08x10e-04	0.0031	0.05	0.06	A	0.26
	SSC6	16	ASGA0089937	120.2-136.0	1.01x10e-05	0.00097	0.0015	-0.07	A	0.35
	SSC7	29	ALGA0041314	50.3-52.4	2.88x10e-04	0.0031	0.04	0.06	A	0.42
	SSC10	1	SIRI0001003	68.0	8.99x10e-05	0.0027	0.01	-0.06	C	0.28

^1^N: Number of significant SNPs, CHR: chromosome, SNP: most significant SNP, Reg (Mb): region containing significant SNPs according to Ensembl (S.scrofa 10.2), *P*: nominal *P*-value, q: q-value with FDR ≤ 0.05, Bonf: Bonferroni-corrected P-value, A1: minority allele, MAF: frequency of the minority allele, E: allelic effect.

Analysis of the gene content of TARs detected with mixed-model methods (Additional file 3: Table S2, Additional file 4: Table S3 and Additional file 5: Table S4) allowed detecting loci related to a variety of lipid metabolic pathways such as cholesterol transport and/or uptake (*ABCG1, APOB*), lipoprotein clearance (*SDC1*) and regulation of lipid metabolism and energy expenditure (*LEPR/LEPROT*). With PLINK, we detected, amongst others, TARs at SSC3 (124 Mb, CHOL190 and LDL190) and SSC6 (135 Mb, CHOL190 and TRIG190), thus providing an independent confirmation of the results obtained with mixed-model approaches. The list of genes mapping to TARs detected with PLINK (Additional file 6: Table S5) was much larger than the ones obtained with mixed-model methods allowing to perform pathway analyses. In this way, genes were mapped to the Reactome database [21] in order to define the metabolic pathways they belong to. Interestingly, the most significantly enriched pathway was “Metabolism of lipids and lipoproteins” (Additional file 7: Table S6), with a 1.89-fold enrichment and a nominal *P*-value of 0.002 (however, this pathway was not significantly enriched after correction for multiple testing *i.e.* Bonferroni corrected *P*-value = 0.14).

We also compared the GWAS data generated in the current experiment with results produced in a QTL scan in the same resource population with a panel of 109 informative microsatellites [5]. As shown in Table 4, only one region at SSC3 (124 Mb) with effects on CHOL190 and LDL190 showed a perfect positional concordance across the four association analysis packages and the QTL scan [5]. We can conclude that this association is very robust and deserves to be further investigated. We found some additional correspondences between a QTL for LDL45 at SSC13 (104 cM) and a TAR detected with GEMMA at 215 Mb, as well as between CHOL190 and LDL190 QTL found at SSC13 (72-74 cM) and TARs detected with PLINK at SSC13 180-181 Mb and 207-210 Mb regions (Table 4).

**Table 4:** **Positional concordance between QTL detected by Gallardo et al.** [5] **and GWAS data generated in the current study**

SSC	Trait	QTL Peak	GEMMA (Mb)	EMMAX (Mb)	GenABEL (Mb)	PLINK (Mb)
3	CHOL190	72 cM (~122 Mb)	124.8	124.6-124.8	124.8	124.8-138.6
3	LDL190	68 cM (~122 Mb)	124.8	124.8	124.8	124.8-138.9
3	TRIG190	83 cM (~131 Mb)	-	-	-	126.4-126.9
13	LDL45	104 cM (~206 Mb)	215	-	-	-
13	CHOL190	72 cM (~194 Mb)	-	-	-	180.8-181.9
						208.3-210.2
13	LDL190	74 cM (~194 Mb)	-	-	-	180.8-199.5
						207.6-210.4

### Orthologous relationships between pig and human genomic regions associated with serum lipids

We have examined the orthologous relationships between the TARs that displayed the most significant and robust associations with serum lipids, and those previously identified in human GWAS [22, 24, 25]. It was obvious the existence of a tight positional concordance for the *APOB* gene. In pigs, this locus maps to a SSC3 genomic region associated with CHOL190, LDL45 and LDL190 levels in the Lipgen population. In humans, *APOB* variability has been also associated with CHOL and LDL concentrations [24, 25]. Another potential correspondence was observed for a SSC6 region (~135 Mb) associated with CHOL190, that lies close to the angiopoietin-related protein 3 gene (*ANGPTL3*, 138 Mb in SSC6) and the dedicator of cytokinesis protein 7 (*DOCK7*, unmapped in pigs, but in human it co-localizes with *ANGPTL3*). In human, these two loci have been consistently associated with CHOL, LDL and TRIG levels [24, 25]. We also detected a third correspondence between the HDL45 TAR at SSC18 (20-22 Mb), exclusively detected with GenABEL, and one region at human 7q32 that contains a microRNA-encoding gene (miR-29A) strongly associated (1 × 10^-15^) with HDL levels [24]. With regard to the most significant PLINK TARs (Table 3), we found some additional orthologous relationships. Near the SSC1 (264-271 Mb) TAR, associated with CHOL190, there is the *ABCA1* gene, strongly associated with HDL-cholesterol in humans [24, 25]. Besides, the MOSC1 gene, that in humans displays associations with LDL levels [24, 25] was located within the SSC10 (0.9-16 Mb) TAR for TRIG45 concentrations

### Search of associations between SNPs within TARs and liver gene expression phenotypes

With the aim of gaining additional insights into the molecular basis of the associations found, we have investigated if 507 SNPs mapping to TARs identified with GEMMA are also associated with gene expression phenotypes. The most significant associations are depicted at Table 5 and the full dataset can be found at Additional file 8: Table S7. Only one association (CHOL45 TAR SNPs at SSC16 vs *MRFAP1* mRNA levels) was significant after applying the Bonferroni correction for multiple testing. Several of the genes whose expression was suggestively associated with TAR SNPs play a significant role in lipid metabolism. Amongst these, we would like to mention the poly (ADP-ribose) polymerase 2 (*PARP2*) locus, that maps to SSC7 and whose expression levels are associated with the SSC13 TAR. Other loci of interest were the synaptonemal complex protein 3 (*SYCP3*) gene, the CDGSH iron sulfur domain 2 (*CISD2*) gene, and the dipeptidyl-peptidase 4 (*DPP4*) gene.

**Table 5:** **List of the most significant associations between SNP variation at SSC3, SSC6, SSC10 and SSC13 trait-associated regions and hepatic gene expression (the Bonferroni threshold of significance for multiple testing was 1.16 × 10e-7)** ^**1**^

TAR	CHR	N	SNP	Reg (Mb)	Gene name	Gene location	***P***	E	MAF
**SSC3** CHOL190 LDL45 LDL190	3	1	H3GA0010701	125.8	*SLC26A11*	SSC12(2.1 Mb)	8.50 x 10e-07	-0.32	0.13
		1	MARC0072051	129.7	*NSD1*	SSC2(82.1 Mb)	1.64 x10e-06	-0.46	0.10
		1	ASGA0016612	132.6	*FLNB*	SSC13(43.8 Mb)	1.95 x 10e-06	-0.73	0.13
		2	ALGA0021353	129.2	*EXT1*	SSC4(22.0 Mb)	3.51 x 10e-06	-1.59	0.34
		1	DIAS0001347	126.2	*ME1*	SSC1(93.2 Mb)	6.7E x 10e-06	0.26	0.38
		2	MARC0020268	132.7-132.8	*WDR91*	SSC18(14.8 Mb)	6.43 x 10e-06	-0.33	0.20
**SSC6** CHOL190 TRIG190	6	3	INRA0022506	135.0-135.1	*SYCP3*	SSC5(86.4 Mb)	3.29 x 10e-06	-1.10	0.38
		4	ASGA0083694	10.2-10.5	*DCTPP1*	SSC3(18.3 Mb)	2.55 x 10e-06	0.10	0.29
		1	ALGA0116014	10.6	*CNN1*	SSC2(70.5 Mb)	4.62 x 10e-06	-1.35	0.12
**SSC10** LDL45 CHOL45	10	1	ALGA0104304	2.8	*SUPT7L*	SSC3(118.4 Mb)	3.20 x 10e-07	-1.78	0.06
		10	ASGA0046816	18.0-19.2	*NUP210*	SSC13(78.6 Mb)	4.05 x 10e-06	-0.44	0.14
		2	ALGA0056299	2.7-3.1	*EMC3*	SSC13(73.2 Mb)	1.39 x 10e-06	3.57	0.08
		1	ASGA0094765	19.2	*DPP4*	SSC15(76.1 Mb)	1.73 x 10e-06	-0.28	0.04
					*UBQLN1*	SSC10(35.3 Mb)	3.18 x 10e-06	-0.27	0.04
					*IFT46*	SSC9(50.8 Mb)	3.33 x 10e-06	-4.5	0.04
		1	MARC0098797	18.8	*CISD2*	SSC8(127.1 Mb)	5.42 x 10e-06	-3.2	0.08
SSC13 LDL45	13	1	ALGA0074022	215.0	*PARP2*	SSC7(83.5 Mb)	1.50 x 10e-07	-2.7	0.08
					*ZNF280C*	SSCX(122.5 Mb)	2.50 x 10e-07	-2.3	0.08
SSC16 CHOL45	16	1	ASGA0072378	17.7	*MRFAP1*	SSC8(3.1 Mb)	8.00 x 10e-08	-0.19	0.12
					*QSOX1*	SSC9(133.5 Mb)	5.60 x 10e-006	-0.41	0.12

^1^TAR: trait-associated region, CHR: chromosome, N:number of significant SNPs, Reg (Mb): region containing significant SNPs according to Ensembl (S.scrofa 10.2), *P*: Nominal P-value, E: allelic effect, MAF: frequency of the minority allele.

## Discussion

### Existence of missing heritability for porcine serum lipid traits

As shown in Table 1, the amount of phenotypic variance explained by the genotyped SNPs was in general lower than heritability values described by Casellas et al. [11] in the same population. This phenomenon of “missing heritability” has been frequently described in GWAS studies. In particular, GWAS are short on their ability to identify rare variants with small effects over the phenotype, which might be the case of many traits of polygenic architecture. One additional limiting factor of GWAS studies performed in livestock is that sample sizes are usually much smaller than those employed in humans. Although the size of our Duroc population is comparable to those described in previous porcine GWAS studies [26–28], the detection of loci with small effects or rare variants with strong effects might be feasible only with larger sample sizes. Despite this limitation, much larger studies performed in humans (in the order of 60,000-100,000 individuals) are consistent with the data outlined in our work. For instance, Asselbergs et al. [29] carried out a meta-analysis of 32 GWAS encompassing 50,000 SNP markers and 66,240 European individuals and found that the proportion of phenotypic variance attributable to the genotyped SNP was 10.3% for CHOL, 9.9% for HDL, 9.5% for LDL and 8.0% for TRIG. Similarly, Teslovich et al. [24] demonstrated that around 25-30% of the genetic variance of plasma lipids could be explained by the variation of SNPs located at 95 loci. Failure to detect additional sources of genetic variance can have multiple causes. For instance, commercial genotyping arrays might contain neither all common nor all rare variants with moderate to large effects on the trait under analysis, so these alleles will be systematically missed in GWAS studies (unless they are in linkage disequilibrium with one or several markers of the array). This can be especially problematic if there is ascertainment bias *i.e.* populations used to build the array are distantly related to the one being studied. Imprecise phenotyping, improper statistical analyses and ignoring other sources of genetic variability (*e.g.* structural variation) can also mask part of V_G_.

The amount of V_P_ explained by the SNPs for HDL45 (0%) and HDL190 (2%) was very low. This observation is coherent with the small Bayes factors (BF) obtained by Casellas et al. [11], in the same Duroc population, when comparing two models with and without additive polygenic effects *i.e.* BF = 2.2. and 2.1 for HDL45 and HDL190, respectively. Such results, according to the scale of Jeffreys [30], are barely worth mentioning. In strong contrast, Bayes factors for CHOL190, LDL190, TRIG45 and TRIG190 ranged between 8.7-47.9 (substantial to very strong evidence favoring the model with polygenic effects). These results imply that the genetic determinism of HDL45 and HDL190 in the Lipgen population is much weaker than that of other serum lipid traits, or that the genetic architecture of these two traits relies on a large amount of loci with very small effects that cannot be captured efficiently with the experimental design and methods used in the current work.

### Genetic determinants of porcine serum lipids are modulated by age-specific factors

Identifying TARs for blood lipid concentrations is particularly difficult because their genetic architecture consists of hundreds of genetic determinants with small effect sizes [24, 25]. The discovery of these TARs, in humans, requires population sizes of tens or even hundreds of thousands of individuals that are unavailable in non-model organisms as pigs. Pigs are particularly interesting because of their physiological similarity with humans and the relative easiness with which tissue samples can be retrieved to analyse gene expression in different experimental conditions. The main trend that emerges from the inspection of data presented at Table 2 is the complete lack of concordance between genotype-phenotype associations detected in 45- and 190-days-old Duroc pigs (Tables 2 and 3 and Additional file 2: Table S1). Moreover, we have observed important differences in h^2^_SNP_ estimates obtained in 45 days and 190 days-old pigs (in general, older pigs have higher values), as shown at Table 1 and Additional file 1: Figure S1. This result may indicate that the genetic architecture of porcine serum lipids traits is modulated by age-specific factors.

Classical studies performed in humans support this latter conclusion. In a longitudinal study [10], it was shown that heritability estimates were relatively constant across generations, but the expression patterns of genes affecting CHOL, LDL, HDL and TRIG were different in adolescent and middle-aged people *e.g.* only 46% (TRIG) to 80% (CHOL) of the genetic variance was shared by both age groups. Indeed, heritability estimates of age-related variations in LDL (h^2^ = 0.25-0.36) and HDL (h^2^ = 0.23-0.58) concentrations are moderate [31], meaning that the relative contributions of their genetic determinants change over time. Even more, a comparison of GWAS data obtained in young and adult people revealed that no single association was significant in both groups [32], implying that age is an important modifier in the genetic determinism of circulating lipids.

### Three genomic regions in SSC3, SSC6 and SSC16 display consistent associations with porcine serum lipid concentrations

There are three regions at SSC3 (~124 Mb, associated with LDL45, CHOL190 and LDL190), SSC6 (~135 Mb, CHOL190, TRIG190) and SSC16 (~17 Mb, CHOL45) that were consistently detected with GEMMA, EMMAX and GenABEL, while several others were method-specific (Table 2). This substantial concordance was, to a certain extent, unexpected because Zhou and Stephens [15] showed that, in the presence of a marked sample structure, approximate methods tend to underestimate *P*-values (*i.e*. they are less significant) and involve a substantial loss of power. Although in general nominal and corrected *P*-values obtained with GEMMA were more significant than those retrieved with EMMAX and GenABEL (Table 2), we did not see neither important *P*-value departures among methods nor a poorer performance of GenABEL (in generating deflated *P*-values) when compared with EMMAX. It is also true, however, that GEMMA was the method that yielded more method-specific associations (CHOL 45 at SSC3 and SSC10, LDL45 at SSC10 and SSC13, TRIG 190 at two SSC6 regions), something that might be explained by an increase in statistical power associated with the performance of exact instead of approximate significance tests.

Genome-wide association analyses carried out with PLINK [15] identified four of the most significant TARs also found with mixed-model methods, plus a large list of additional TARs. We believe these differences are explained by the fact that PLINK assumes a completely different approach to handle population structure [15]. Instead of capturing infinitesimal polygenic effects, PLINK relies on standard linear models where family-related effects (*i.e.* sire-mean-adjusted) must be accounted for by appropriate regression coefficients. Alternatively, some specific tests are available for case-control studies when population stratification has been previously identified, although they can not be generalized to quantitative traits [33]. Given that our analyses focused on non-discrete traits, potential population structure was partially accounted for by including sire-specific effects into the linear model (without considering dam-related contributions). This was mainly due to the limitations of the PLINK program to take into account infinitesimal additive genetic effects under non-homogeneous covariance structures, and the fact that sow-related contributions could not be addressed when a single offspring was retained from each litter. Although the inclusion of sire-specific effects in the model must be viewed as a reasonable way to account for hidden population structure in the Lipgen population, results must be taken with caution given the risk of false positives linked to partially undetected sample structure [34].

Analysis of the gene content of genomic 1 Mb-windows around each one of the most significant SNPs within each one of the TARs detected with mixed-model methods revealed the presence of several loci involved in lipid metabolism. As previously said, one of the most promising candidate genes is apolipoprotein B (*APOB*, located at SSC3 125.2 Mb), which has been identified in our study as well as in the GWAS performed by Chen et al. [9]. Apolipoprotein B is essential for the correct assembly of chylomicrons and the synthesis of very low density lipoproteins (VLDL), that transport TRIG from the intestine to other body tissues [35]. Meanwhile, VLDL become progressively lipolyzed into LDL. Since APOB mediates the binding and endocytosis of LDL by their receptors, the knockout of this gene translates into hypercholesterolemia [35]. Close to *APOB,* there is also the syndecan 1 gene (*SDC1,* located at SSC3 125.9 Mb) that encodes a membrane proteoglycan that mediates the clearance of TRIG-rich lipoproteins [36].

The SSC6 region (peak SNP at ~135 Mb) associated with CHOL190 contains the leptin receptor (*LEPR*) and the leptin receptor overlapping transcript (*LEPROT*) genes, both mapping to 135.3 Mb. Leptin plays key roles in (i) the regulation of food intake and energy expenditure, (ii) the modulation of APOB levels and triglyceridemia and (iii) the intestinal absorption of cholesterol [37, 38]. Finally, it is worth to mention the ATP-binding cassette sub-family G (WHITE), member 1 (*ABCG1*), that maps to SSC13 (215.8 Mb) and controls tissue lipid levels and the efflux of cellular cholesterol to HDL.

The list of genes within TARs detected with PLINK was very large (Additional file 6: Table S5), so we mapped them to the Reactome database [21] to achieve a global view of their biological functions. Loci mapping to TARs identified with PLINK and comprised within the “Metabolism of lipids and lipoproteins” Reactome category encompassed genes related with a variety of processes such as lipid transport (*APOA1, APOA4, APOB, APOC3, ABCB11, SCP2*) and clearance (*SDC1*), cholesterol synthesis (*DHCR24, CH25H*), fatty acid β-oxidation (*ACOX1, ACADM*) and phospholipid synthesis (*AGPAT5*).

### Positional concordance for GWAS and QTL data generated in the Lipgen population

We have compared our GWAS data with QTL previously reported by Gallardo et al. [5] in the same population. Regarding mixed-model methods, the most prominent coincidence was a SSC3 region containing chromosome-wide QTL for CHOL190, LDL190 and TRIG190 [5]. The QTL peak at marker SW2408 (approximately 122 Mb) matched TARs for CHOL190, LDL45 and LDL190 (SSC3, ~124 Mb, confirmed with the three programs). Remarkably, Chen et al. [9] identified the same TAR as significantly associated with CHOL and LDL concentrations in F_2_ Erhualian x Duroc pigs. This specific region contains the *APOB* gene that in GWAS studies performed in humans has been consistently associated with CHOL and LDL plasma levels. Apolipoprotein B is the main structural component of chylomicrons and very-low density lipoproteins (VLDL, the precursor of LDL) and plays an essential role in TRIG homeostasis [39]. Interestingly, Pena et al. [40] genotyped a polymorphic 230 bp-intronic insertion at the pig *APOB* gene in the Lipgen population and reported associations with CHOL190, HDL190 and LDL190 concentrations. Taken together, these results suggest that *APOB* genotype might be a major determinant of CHOL and lipoprotein levels both in humans and pigs.

We also observed some concordance between a QTL for LDL45 at SSC13 (104 cM) and a TAR detected with GEMMA at 215 Mb, as well as between CHOL190 and LDL190 QTL found at SSC13 (72-74 cM) and TARs detected with PLINK at the 180-181 Mb and 207-210 Mb regions (Table 4). The existence of a genetic determinant for serum lipids on SSC13 is supported by results from previous genome scans, where QTL for CHOL (SSC13, 212 Mb approx.) and LDL (SSC13, 194 Mb) were detected by Yoo et al. [8] and Uddin et al. [7], respectively.

The limited concordance of QTL scan [5] and GWAS data obtained from the Lipgen population may be explained by differences in marker density, type of polymorphisms and statistical methods to carry out genome-wide analyses. For instance, the analysis of a Chinese Erhualian × White Duroc three generation population yielded QTL [6] and TAR [9] maps that were remarkably different *i.e* in the GWAS the main associations mapped to SSC1 (63 Mb, LDL) and SSC3 (124 Mb, CHOL and LDL); whilst in the QTL scan SSC2 (67-73 cM, CHOL, LDL and TRIG), SSC5 (70 cM, TRIG), SSC7 (134 cM, HDL) and SSC8 (87 cM, LDL) encompassed the most significant associations. Similarly, Ramayo-Caldas et al. [26] reported that only 53% of the TARs detected in their GWAS study coincided with previously reported porcine QTL.

### Evidences of positional concordance between trait-associated regions in humans and pigs

Gallardo et al. [5] reported that there is a remarkable level of correspondence between lipid QTL found in human and pigs. However, the resolution of this study was severely limited by the fact that QTL intervals were defined on the basis of 109 microsatellites spaced approximately every 20 cM. Comparison of orthologous relationships between TARs generated in our study and those published in the NHGRI GWAS Catalog [22] revealed few concordances. The most obvious one affected the *APOB* gene, that maps to SSC3 (125 Mb) and Hsa2 (21 Mb) in pigs and humans, respectively. In the study of Teslovich et al. [24], this locus showed pleiotropic effects on the lipid profile, being highly associated (4 × 10^-114^) with cholesterol and LDL levels. Another potential correspondence was detected for *ANGPTL3* [24] and *DOCK7* [25]. Loss-of-function mutations in the *ANGPTL3* gene are known to be associated with decreased levels of LDL, HDL and TRIG [41]. The associations observed for the *DOCK7* locus, which is involved in neurogenesis, myelination and axon formation [42] but not in lipid metabolism, probably reflect the co-localization of this gene with *ANGPTL3*. The *ABCA1* gene also lies close to the SSC1 (264-271 Mb) TAR for CHOL190 (only detected with PLINK), a result that makes sense from a biological point of view because this gene has a major role in cholesterol homeostasis [43].

There are several considerations that need to be taken into account to explain the limited concordance between human and porcine TARs. First, our Duroc commercial line is by no means representative of the whole porcine diversity, so it is quite possible that the analysis of further swine populations might uncover additional orthologous associations with human. Besides, complex traits are known to have a considerable degree of genetic heterogeneity. A recent review highlighted that the level of correspondence between TARs observed in East Asians and Europeans, two populations that diverged 23 kya ago, ranged between 32-100% with a mean of 65% [44]. Moreover, a significant part of these shared European-East Asian associations was explained by different SNP. Since human and pigs diverged around 94 MYR ago [45] it is reasonable to infer that the level of concordance of GWAS signals between species must be necessarily much lower.

### Variation within several TARs is associated with the hepatic expression of lipid metabolism genes

We have discussed the genomic distribution and gene content of blood lipid TARs detected in a Duroc commercial line. Moreover, we have analysed the positional concordance of these TARs with previous data reported in pigs and in humans. In order to gain additional insights into the mechanisms that may explain the associations found, we have examined if SNPs mapping to TARs are also associated with hepatic gene expression levels. Indeed, in a recent study Nicolae et al. [46] concluded that TARs are mostly explained by the segregation of expression QTL (eQTL), thus suggesting that causal mutations exert their effects mainly through the regulation of gene expression. This approach allowed us identifying several genes related to lipid metabolism, that deserve to be further explored (Table 5). For instance, SNPs within the SSC13 TAR for LDL45 were also associated with *PARP2* mRNA expression (nominal *P*-value = 1.50 × 10e-07). Interestingly, the deletion of this gene leads to an increase in the accumulation of cholesterol in the liver by enhancing SREBP1 expression [47]. Other genes of interest were *SLC19A1*, that in humans is associated with HDL levels [48]; *SYCP3*, whose knockdown affects the expression of genes related to lipid metabolism [49]; *CISD2*, that inhibits muscle fat infiltration [50]; and *DPP4*, a gene that is overexpressed in the visceral fat of severely obese individuals [51]. All of these associations involved *trans*-effects, where SNPs within TARs affect the expression of loci mapping to distant locations. According to Cheung et al. [52], *trans*-eQTL are more abundant than those with *cis*-effects and they often involve interactions mediated by molecules other than transcription factors.

## Conclusions

The approach we have employed, based on the combined use of distinct statistical packages, has been successful at identifying several regions of the pig genome (SSC3, SSC6 and SSC16) with robust and significant effects on serum lipid concentrations. Importantly, we have demonstrated that TARs identified at 45 and 190 days do not show positional concordance, a feature that suggests that the effects of causal mutations regulating porcine serum lipid concentrations are modulated by age-specific factors. Several SNPs within TARs are associated with the expression of lipid metabolism genes, suggesting that causal effects may have a regulatory basis. Exploring the genetic diversity of serum lipids in pigs and other non-model species may pave the way to the discovery of novel genes and functions regulating the susceptibility to cardiovascular diseases in humans.

## Availability of supporting data

All the supporting data are included as additional files.

## Electronic supplementary material

Additional file 1: Figure S1: Estimating the proportion of phenotypic variance of serum lipid traits explained by the genotyped SNPs (h^2^_SNP_) by using three different mixed-model based methods. **Figure S2.** Manhattan plots depicting the associations between pig chromosomes 3, 6 and 16 and serum lipid concentrations, as detected with GEMMA (dotted lines indicate the threshold of significance at P = 0.05). (DOC 92 KB)

Additional file 2: Table S1: Results of the PLINK analysis after correcting for population structure (the sire effect is introduced in the statistical model). (DOC 230 KB)

Additional file 3: Table S2: Genes mapping to TARs identified with EMMAX. (XLS 31 KB)

Additional file 4: Table S3: Genes mapping to TARs identified with GenABEL. (XLS 104 KB)

Additional file 5: Table S4: Genes mapping to TARs identified with GEMMA. (XLS 150 KB)

Additional file 6: Table S5: Genes mapping to TARs identified with PLINK. (XLS 2 MB)

Additional file 7: Table S6: Mapping of the genes located at TARs identified with PLINK to the Reactome database. (XLS 10 KB)

Additional file 8: Table S7: List of loci whose expression is nominally associated with SNPs located within TARs. (XLS 44 KB)

## Abbreviations

BF: Bayes Factor
CHOL: Cholesterol
cM: CentiMorgan
EMMAX: Efficient mixed-model eXpedited
GEMMA: Genome-wide efficient mixed-model association
GRAMMAR: Genome-wide rapid association using mixed-model and regression
GWAS: Genome-wide association study
HDL: High density lipoproteins
LDL: Low density lipoproteins
Mb: Megabase
QTL: Quantitative trait locus
SNP: Single nucleotide polymorphism
SSC: Pig chromosome
TAR: Trait-associated region
TRIG: Triglycerides.
