# Supplementary material for: A genome-wide association analysis for porcine serum lipid traits reveals the existence of age-specific genetic determinants
Source: BMC Genomics. 2014 Sep 4;15(1):758. doi: 10.1186/1471-2164-15-758 (PMC4164741; doi:10.1186/1471-2164-15-758)

Additional file 1: Figure S1. Estimating the proportion of phenotypic variance of serum lipid traits explained by the genotyped SNPs (h^2^_SNP_) by using three different mixed-model based methods.

Figure S2. Manhattan plots depicting the associations between pig chromosomes 3, 6 and 16 and serum lipid concentrations, as detected with GEMMA.


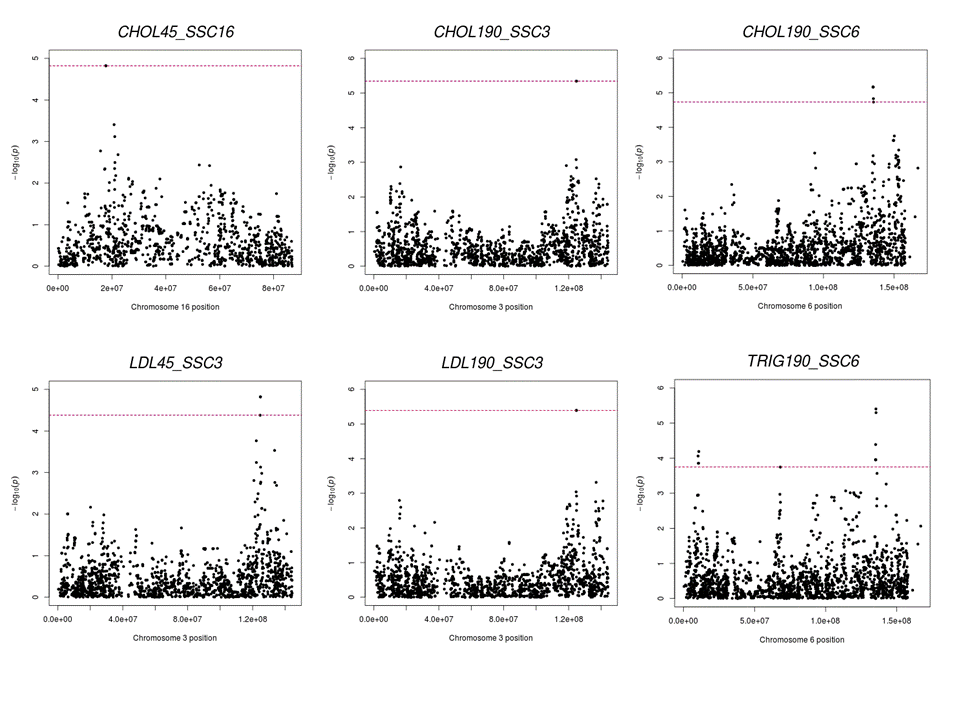

Supplement: Supplementary file 1 — Additional file 1: Figure S1: Estimating the proportion of phenotypic variance of serum lipid traits explained by the genotyped SNPs (h2 SNP) by using three different mixed-model based methods. Figure S2. Manhattan plots depicting the associations between pig chromosomes 3, 6 and 16 and serum lipid concentrations, as detected with GEMMA (dotted lines indicate the threshold of significance at P = 0.05). (DOC 92 KB) [file 12864_2013_6435_MOESM1_ESM.doc]
