# Supplementary material for: A genome-wide association analysis for porcine serum lipid traits reveals the existence of age-specific genetic determinants
Source: BMC Genomics. 2014 Sep 4;15(1):758. doi: 10.1186/1471-2164-15-758 (PMC4164741; doi:10.1186/1471-2164-15-758)
Supplement: Supplementary file 2 — Additional file 2: Table S1: Results of the PLINK analysis after correcting for population structure (the sire effect is introduced in the statistical model). (DOC 230 KB) [file 12864_2013_6435_MOESM2_ESM.doc]

Supplementary Table 1. Results of the PLINK analysis after correcting for population structure (the sire effect is introduced in the statistical model).

| LDL190 | CHR | N | SNP | START | STOP | P | q | Bonf | A1 | Freq | E |
| --- | --- | --- | --- | --- | --- | --- | --- | --- | --- | --- | --- |
|  | 1 | 1 | ASGA0089858 | 14171701 | 14171701 | 1.04x10e-03 | 0.01 | 0.24 | A | 0.20 | 0.04 |
|  | 1 | 50 | ALGA0001735 | 24454868 | 28728265 | 8.95x10e-04 | 0.01 | 0.20 | A | 0.20 | 0.04 |
|  | 1 | 1 | ALGA0009612 | 287428107 | 287428107 | 2.21x10e-02 | 0.03 | 1.00 | A | 0.50 | 0.02 |
|  | 1 | 1 | MARC0010692 | 302552307 | 302552307 | 5.40x10e-03 | 0.01 | 1.00 | A | 0.28 | -0.03 |
|  | 2 | 1 | H3GA0006199 | 19419594 | 19419594 | 8.17x10e-03 | 0.02 | 1.00 | A | 0.37 | -0.03 |
|  | 3 | 2 | H3GA0055069 | 10137251 | 16029880 | 7.84x10e-03 | 0.02 | 1.00 | G | 0.13 | -0.04 |
|  | 3 | 13 | ALGA0021216 | 124818275 | 138956364 | 1.46x10e-04 | 0.01 | 0.03 | A | 0.33 | -0.04 |
|  | 3 | 2 | ALGA0021769 | 141134216 | 141168792 | 3.43x10e-03 | 0.01 | 0.78 | G | 0.11 | 0.05 |
|  | 4 | 1 | H3GA0012847 | 69896058 | 69896058 | 1.01x10e-02 | 0.02 | 1.00 | A | 0.17 | -0.04 |
|  | 6 | 7 | ALGA0036903 | 126645776 | 135188378 | 4.46x10e-04 | 0.01 | 0.10 | A | 0.35 | 0.04 |
|  | 6 | 3 | MARC0041415 | 141940557 | 146304375 | 1.16x10e-03 | 0.01 | 0.26 | A | 0.48 | 0.03 |
|  | 7 | 2 | MARC0075038 | 11321769 | 15995030 | 7.42x10e-03 | 0.02 | 1.00 | G | 0.09 | 0.05 |
|  | 7 | 1 | ALGA0042294 | 63745647 | 63745647 | 2.45x10e-02 | 0.04 | 1.00 | G | 0.37 | -0.03 |
|  | 7 | 1 | ASGA0036089 | 117195566 | 117195566 | 1.89x10e-03 | 0.01 | 0.43 | G | 0.22 | 0.04 |
|  | 8 | 10 | ALGA0104210 | 7875856 | 15992070 | 5.78x10e-03 | 0.01 | 1.00 | C | 0.11 | -0.04 |
|  | 8 | 1 | ALGA0108646 | 133819839 | 133819839 | 1.67x10e-02 | 0.03 | 1.00 | G | 0.33 | -0.03 |
|  | 8 | 1 | ASGA0092699 | 145089204 | 145089204 | 8.07x10e-03 | 0.02 | 1.00 | A | 0.44 | 0.03 |
|  | 9 | 1 | ASGA0041847 | 16129184 | 16129184 | 1.93x10e-02 | 0.03 | 1.00 | A | 0.31 | -0.03 |
|  | 9 | 1 | DIAS0000699 | 55098439 | 55098439 | 1.35x10e-02 | 0.03 | 1.00 | G | 0.27 | 0.03 |
|  | 10 | 1 | ALGA0116145 | 30978174 | 30978174 | 1.75x10e-02 | 0.03 | 1.00 | A | 0.45 | 0.02 |
|  | 10 | 6 | ASGA0096596 | 40950374 | 53939074 | 1.31x10e-02 | 0.02 | 1.00 | G | 0.44 | -0.03 |
|  | 10 | 4 | ALGA0103072 | 69856206 | 76905575 | 3.62x10e-04 | 0.01 | 0.08 | G | 0.31 | 0.04 |
|  | 12 | 1 | ASGA0053505 | 16784171 | 16784171 | 2.06x10e-02 | 0.03 | 1.00 | A | 0.46 | -0.03 |
|  | 13 | 1 | MARC0029581 | 15811761 | 15811761 | 3.08x10e-02 | 0.04 | 1.00 | G | 0.46 | -0.02 |
|  | 13 | 1 | ALGA0071516 | 100946188 | 100946188 | 1.94x10e-02 | 0.03 | 1.00 | G | 0.26 | -0.03 |
|  | 13 | 8 | MARC0015993 | 154021170 | 157429895 | 1.38x10e-02 | 0.03 | 1.00 | A | 0.47 | -0.03 |
|  | 13 | 3 | ALGA0072740 | 175053396 | 178659261 | 5.78x10e-03 | 0.01 | 1.00 | G | 0.50 | 0.03 |
|  | 13 | 3 | MARC0016316 | 180877890 | 199554583 | 2.23x10e-03 | 0.01 | 0.51 | A | 0.48 | -0.03 |
|  | 13 | 7 | ALGA0109869 | 207625341 | 210415823 | 5.61x10e-03 | 0.01 | 1.00 | G | 0.31 | 0.03 |
|  | 15 | 3 | MARC0053020 | 26551247 | 39256217 | 5.02x10e-03 | 0.01 | 1.00 | G | 0.14 | -0.04 |
|  | 15 | 10 | MARC0043488 | 40780676 | 59799712 | 2.62x10e-03 | 0.01 | 0.59 | A | 0.18 | -0.05 |
|  | 15 | 1 | H3GA0044381 | 60008146 | 60008146 | 1.55x10e-02 | 0.03 | 1.00 | G | 0.17 | -0.05 |
|  | 15 | 9 | MARC0049283 | 84133242 | 86545656 | 1.30x10e-03 | 0.01 | 0.30 | A | 0.31 | -0.04 |
|  | 17 | 1 | ASGA0075426 | 10789252 | 10789252 | 8.31x10e-04 | 0.01 | 0.19 | G | 0.43 | 0.04 |
|  | 17 | 3 | ASGA0075693 | 21985990 | 31375457 | 1.45x10e-02 | 0.03 | 1.00 | A | 0.43 | -0.03 |
|  | 18 | 1 | ASGA0094270 | 4997045 | 4997045 | 4.16x10e-03 | 0.01 | 0.94 | G | 0.34 | 0.03 |
| CHOL190 | 1 | 2 | ASGA0089858 | 11037437 | 14171701 | 1.24x10e-03 | 0.01 | 0.28 | A | 0.20 | 0.03 |
|  | 1 | 49 | ALGA0001735 | 24454868 | 28728265 | 1.08x10e-03 | 0.01 | 0.25 | A | 0.20 | 0.03 |
|  | 1 | 6 | M1GA0001375 | 264617769 | 271950519 | 1.49x10e-04 | 0.004 | 0.03 | A | 0.39 | 0.03 |
|  | 1 | 3 | ASGA0006863 | 283832963 | 284552738 | 2.81x10e-03 | 0.01 | 0.64 | G | 0.45 | -0.02 |
|  | 1 | 1 | MARC0010692 | 302552307 | 302552307 | 7.69x10e-03 | 0.02 | 1.00 | A | 0.28 | -0.02 |
|  | 2 | 4 | ALGA0011877 | 10372649 | 10652052 | 4.47x10e-04 | 0.01 | 0.10 | A | 0.40 | 0.03 |
|  | 2 | 2 | ALGA0106794 | 28105295 | 28301844 | 7.24x10e-04 | 0.01 | 0.17 | G | 0.24 | 0.03 |
|  | 3 | 4 | MARC0063508 | 10137251 | 16531401 | 3.06x10e-03 | 0.01 | 0.70 | C | 0.17 | -0.03 |
|  | 3 | 5 | ALGA0021216 | 124818275 | 138684370 | 2.84x10e-04 | 0.01 | 0.06 | A | 0.33 | -0.03 |
|  | 4 | 1 | H3GA0012847 | 69896058 | 69896058 | 1.17x10e-02 | 0.02 | 1.00 | A | 0.17 | -0.02 |
|  | 5 | 1 | ALGA0033296 | 89146554 | 89146554 | 7.62x10e-03 | 0.02 | 1.00 | A | 0.28 | 0.02 |
|  | 6 | 1 | ALGA0110498 | 93783397 | 93783397 | 1.42x10e-04 | 0.004 | 0.03 | A | 0.12 | -0.04 |
|  | 6 | 7 | ALGA0037119 | 135074196 | 136241531 | 4.86x10e-05 | 0.004 | 0.01 | G | 0.36 | 0.03 |
|  | 6 | 14 | ASGA0030240 | 145998261 | 153491491 | 9.02x10e-05 | 0.003 | 0.02 | A | 0.32 | 0.03 |
|  | 6 | 1 | MARC0058173 | 166855362 | 166855362 | 4.91x10e-03 | 0.01 | 1.00 | A | 0.26 | 0.02 |
|  | 8 | 2 | ASGA0092178 | 14946506 | 18822892 | 5.27x10e-03 | 0.01 | 1.00 | G | 0.33 | 0.02 |
|  | 8 | 1 | ALGA0049517 | 105795166 | 105795166 | 2.05x10e-02 | 0.04 | 1.00 | G | 0.26 | -0.02 |
|  | 8 | 4 | ALGA0108646 | 130050222 | 133819839 | 8.04x10e-03 | 0.02 | 1.00 | G | 0.33 | -0.02 |
|  | 8 | 1 | ASGA0092699 | 145089204 | 145089204 | 7.46x10e-03 | 0.02 | 1.00 | A | 0.44 | 0.02 |
|  | 9 | 1 | ASGA0041847 | 16129184 | 16129184 | 8.05x10e-03 | 0.02 | 1.00 | A | 0.31 | -0.02 |
|  | 9 | 1 | MARC0014181 | 41839457 | 41839457 | 1.78x10e-02 | 0.03 | 1.00 | C | 0.29 | -0.02 |
|  | 10 | 3 | ASGA0047452 | 27292836 | 39058913 | 1.77x10e-03 | 0.01 | 0.40 | G | 0.18 | 0.03 |
|  | 10 | 2 | ALGA0103072 | 76863103 | 76905575 | 3.74x10e-04 | 0.01 | 0.09 | G | 0.31 | 0.03 |
|  | 13 | 5 | ALGA0109098 | 154864732 | 155429333 | 2.22x10e-02 | 0.04 | 1.00 | A | 0.38 | 0.02 |
|  | 13 | 6 | CASI0009496 | 170870787 | 178659261 | 5.10x10e-03 | 0.01 | 1.00 | A | 0.30 | 0.03 |
|  | 13 | 2 | MARC0016316 | 180877890 | 181995091 | 4.79x10e-03 | 0.01 | 1.00 | A | 0.48 | -0.02 |
|  | 13 | 5 | MARC0058120 | 208316160 | 210294458 | 7.59x10e-03 | 0.02 | 1.00 | A | 0.48 | 0.02 |
|  | 15 | 5 | MARC0043488 | 59388666 | 59799712 | 2.95x10e-03 | 0.01 | 0.67 | A | 0.18 | -0.04 |
|  | 15 | 6 | H3GA0044381 | 60008146 | 60719311 | 1.83x10e-02 | 0.03 | 1.00 | G | 0.17 | -0.03 |
|  | 15 | 2 | ASGA0069883 | 86048980 | 86545656 | 3.29x10e-02 | 0.05 | 1.00 | A | 0.24 | -0.02 |
|  | 17 | 1 | ASGA0075426 | 10789252 | 10789252 | 3.34x10e-03 | 0.01 | 0.76 | G | 0.43 | 0.02 |
|  | 17 | 2 | ASGA0075693 | 21985990 | 22093068 | 7.40x10e-03 | 0.02 | 1.00 | A | 0.43 | -0.02 |
| TRIG45 | 1 | 2 | ASGA0008038 | 304598157 | 304648272 | 1.10x10e-03 | 0.01 | 0.15 | A | 0.28 | -0.04 |
|  | 2 | 1 | H3GA0052992 | 8379515 | 8379515 | 1.35x10e-02 | 0.02 | 1.00 | A | 0.46 | 0.03 |
|  | 2 | 1 | MARC0001645 | 87106233 | 87106233 | 5.97x10e-04 | 0.01 | 0.08 | A | 0.33 | 0.04 |
|  | 2 | 5 | DIAS0003555 | 144171843 | 147952931 | 3.20x10e-03 | 0.01 | 0.42 | G | 0.33 | 0.05 |
|  | 3 | 1 | ALGA0017462 | 10869998 | 10869998 | 5.31x10e-03 | 0.01 | 0.70 | G | 0.46 | 0.04 |
|  | 3 | 3 | ALGA0019221 | 58814766 | 59884491 | 5.30x10e-03 | 0.01 | 0.70 | A | 0.38 | -0.03 |
|  | 3 | 1 | H3GA0009664 | 60015652 | 60015652 | 5.30x10e-03 | 0.01 | 0.70 | A | 0.38 | -0.03 |
|  | 3 | 7 | ASGA0015301 | 94216284 | 97947203 | 1.90x10e-02 | 0.03 | 1.00 | C | 0.50 | 0.03 |
|  | 3 | 6 | ALGA0021310 | 124647480 | 128410163 | 4.88x10e-03 | 0.01 | 0.64 | A | 0.27 | 0.05 |
|  | 5 | 3 | MARC0085569 | 68124100 | 68417760 | 8.41x10e-03 | 0.02 | 1.00 | G | 0.07 | -0.07 |
|  | 5 | 1 | ALGA0033296 | 89146554 | 89146554 | 7.77x10e-03 | 0.01 | 1.00 | A | 0.28 | 0.04 |
|  | 6 | 1 | ASGA0100039 | 14509618 | 14509618 | 1.27x10e-02 | 0.02 | 1.00 | A | 0.45 | -0.03 |
|  | 7 | 1 | MARC0058691 | 10744544 | 10744544 | 1.73x10e-04 | 0.01 | 0.02 | G | 0.20 | 0.06 |
|  | 7 | 9 | H3GA0022580 | 95616421 | 99992288 | 5.61x10e-04 | 0.01 | 0.07 | G | 0.38 | 0.05 |
|  | 7 | 2 | ALGA0043835 | 100038150 | 100145085 | 4.09x10e-04 | 0.01 | 0.05 | A | 0.38 | 0.05 |
|  | 8 | 2 | ALGA0047098 | 28853891 | 29095960 | 5.22x10e-03 | 0.01 | 0.69 | G | 0.10 | 0.07 |
|  | 8 | 3 | ALGA0048133 | 74879258 | 75648737 | 1.39x10e-02 | 0.02 | 1.00 | A | 0.20 | 0.04 |
|  | 8 | 6 | MARC0025408 | 120734566 | 121026032 | 9.74x10e-03 | 0.02 | 1.00 | A | 0.26 | 0.04 |
|  | 9 | 2 | H3GA0027196 | 48645290 | 48835703 | 1.14x10e-03 | 0.01 | 0.15 | A | 0.25 | 0.05 |
|  | 10 | 7 | MARC0042485 | 933652 | 16173744 | 7.04x10e-06 | 0.001 | 0.001 | A | 0.29 | 0.06 |
|  | 10 | 7 | MARC0003307 | 38009455 | 39007437 | 1.11x10e-04 | 0.01 | 0.01 | A | 0.33 | -0.05 |
|  | 11 | 1 | H3GA0031439 | 16289120 | 16289120 | 4.82x10e-04 | 0.01 | 0.06 | A | 0.30 | -0.05 |
|  | 11 | 1 | H3GA0032530 | 79415461 | 79415461 | 7.91x10e-03 | 0.01 | 1.00 | A | 0.42 | 0.03 |
|  | 14 | 2 | ALGA0074770 | 1845285 | 7521763 | 9.84x10e-03 | 0.02 | 1.00 | A | 0.46 | -0.03 |
|  | 14 | 33 | ALGA0080030 | 101181073 | 114007859 | 2.05x10e-04 | 0.01 | 0.03 | A | 0.14 | -0.07 |
|  | 16 | 4 | ASGA0072378 | 4216600 | 17751141 | 5.29x10e-04 | 0.01 | 0.07 | G | 0.12 | 0.06 |
| TRIG190 | 1 | 4 | ALGA0108388 | 28612780 | 30204123 | 3.08x10e-04 | 0.003 | 0.05 | A | 0.26 | 0.06 |
|  | 1 | 1 | MARC0096953 | 67608660 | 67608660 | 3.85x10e-03 | 0.01 | 0.60 | A | 0.07 | -0.10 |
|  | 2 | 2 | ASGA0009469 | 15840916 | 19544610 | 1.29x10e-02 | 0.02 | 1.00 | G | 0.13 | -0.09 |
|  | 2 | 3 | ASGA0009510 | 20081797 | 21560511 | 9.03x10e-03 | 0.01 | 1.00 | G | 0.21 | -0.06 |
|  | 2 | 1 | H3GA0006745 | 48612084 | 48612084 | 1.84x10e-02 | 0.03 | 1.00 | A | 0.32 | -0.05 |
|  | 3 | 5 | H3GA0008910 | 13192716 | 15772472 | 4.59x10e-03 | 0.01 | 0.71 | G | 0.25 | -0.06 |
|  | 3 | 4 | ALGA0021292 | 126491018 | 126989393 | 6.36x10e-03 | 0.01 | 0.99 | A | 0.28 | 0.06 |
|  | 4 | 1 | H3GA0013586 | 104027961 | 104027961 | 2.15x10e-03 | 0.01 | 0.33 | G | 0.46 | 0.04 |
|  | 5 | 1 | INRA0058339 | 81462986 | 81462986 | 6.27x10e-03 | 0.01 | 0.97 | G | 0.15 | -0.09 |
|  | 6 | 9 | ASGA0101719 | 7408752 | 10774936 | 6.89x10e-05 | 0.003 | 0.01 | G | 0.36 | -0.07 |
|  | 6 | 1 | MARC0039313 | 30873612 | 30873612 | 5.77x10e-03 | 0.01 | 0.89 | A | 0.16 | -0.06 |
|  | 6 | 3 | ASGA0106002 | 67811198 | 68188373 | 9.49x10e-04 | 0.005 | 0.15 | A | 0.43 | -0.06 |
|  | 6 | 15 | ALGA0110498 | 88616811 | 94464352 | 1.38x10e-03 | 0.01 | 0.21 | A | 0.12 | -0.08 |
|  | 6 | 17 | ALGA0036415 | 104186476 | 119815135 | 2.05x10e-03 | 0.01 | 0.32 | G | 0.11 | -0.08 |
|  | 6 | 16 | ASGA0089937 | 120225026 | 136045327 | 1.01x10e-05 | 0.001 | 0.001 | A | 0.35 | -0.07 |
|  | 6 | 3 | ALGA0037404 | 142393332 | 147195355 | 1.92x10e-03 | 0.01 | 0.30 | G | 0.08 | -0.09 |
|  | 7 | 1 | H3GA0020083 | 15171138 | 15171138 | 2.85x10e-03 | 0.01 | 0.44 | G | 0.39 | 0.04 |
|  | 7 | 1 | H3GA0020948 | 38236482 | 38236482 | 3.01x10e-02 | 0.04 | 1.00 | A | 0.32 | -0.04 |
|  | 7 | 29 | ALGA0041314 | 50327355 | 52462626 | 2.88x10e-04 | 0.003 | 0.04 | A | 0.42 | 0.06 |
|  | 7 | 4 | ALGA0042326 | 64721179 | 65695206 | 1.29x10e-03 | 0.01 | 0.20 | C | 0.20 | 0.06 |
|  | 7 | 2 | MARC0058228 | 126014404 | 134388813 | 1.25x10e-03 | 0.01 | 0.19 | C | 0.43 | -0.05 |
|  | 9 | 2 | ALGA0123865 | 132656946 | 132659971 | 7.44x10e-03 | 0.01 | 1.00 | G | 0.10 | -0.07 |
|  | 10 | 2 | ALGA0056709 | 8794033 | 8807402 | 1.08x10e-02 | 0.02 | 1.00 | G | 0.25 | -0.06 |
|  | 10 | 1 | SIRI0001003 | 68087317 | 68087317 | 8.99x10e-05 | 0.003 | 0.01 | C | 0.28 | -0.06 |
|  | 12 | 3 | ALGA0064381 | 4302747 | 8203201 | 1.52x10e-03 | 0.01 | 0.24 | A | 0.29 | -0.07 |
|  | 14 | 2 | M1GA0019680 | 149141428 | 149388611 | 1.95x10e-03 | 0.01 | 0.30 | G | 0.19 | -0.06 |
|  | 15 | 6 | ALGA0110920 | 28323170 | 30265754 | 1.70x10e-02 | 0.02 | 1.00 | G | 0.34 | -0.04 |
|  | 16 | 1 | ASGA0094242 | 80029525 | 80029525 | 1.78x10e-02 | 0.03 | 1.00 | C | 0.07 | -0.08 |

^1^N: Number of significant SNPs, CHR: chromosome, SNP: most significant SNP, Reg (Mb): region containing significant SNPs according to Ensembl (S.scrofa 10.2), p: nominal *P*-value, q: q-value with FDR ≤ 0.05, A1: minority allele, MAF: frequency of the minority allele, E: allelic effect.
